# Extraction, Characterization and Osteogenic Activity of a Type I Collagen from Starfish (*Asterias amurensis*)

**DOI:** 10.3390/md21050274

**Published:** 2023-04-27

**Authors:** Lingcui Li, Yu Yu, Wenhui Wu, Peipei Wang

**Affiliations:** 1 Department of Marine Pharmacology, College of Food Science and Technology, Shanghai Ocean University, Shanghai 201306, China; m200300941@st.shou.edu.cn; 2 Marine Biomedical Science and Technology Innovation Platform of Lin-Gang Special Area, Shanghai 201306, China; 3 Qingdao Institute for Food and Drug Control, Qingdao 266071, China; 8639395@163.com

**Keywords:** starfish, *Asterias amurensis*, type I collagen, osteogenic differentiation

## Abstract

Outbreaks of starfish (*Asterias amurensis*) pose a major threat to aquaculture and marine ecosystems in Qingdao, China, and no effective methods have been found to control them. A comprehensive study of collagen in starfish could be an alternative to high efficient utilization. Based on this, collagen was firstly extracted from Qingdao *A. amurensis*. Then, its protein pattern, amino acid composition, secondary structure, microstructure and thermal stability were investigated. The results showed that the *A. amurensis* collagen (AAC) is a type I collagen composed of α_1_, α_2_, and β chains. Glycine, hydroxyproline, and alanine were the major amino acids. The melting temperature was 57.7 °C. From FTIR, UV spectra and CD chromatography, the AAC had an intact triple helix and secondary structure, and microstructural analysis showed that the AAC had a loose, fibrous porous structure. Next, the osteogenic differentiation effect of AAC on Mouse bone marrow stem cells (BMSCs) was investigated, and the results showed that AAC induced osteogenic differentiation of cells by promoting the proliferation of BMSCs, enhancing alkaline phosphatase (ALP) activity, promoting cell mineralization nodules and upregulating the expression of mRNA of relevant osteogenic genes. These results suggest that AAC might have the potential application to bone health-related functional foods.

## 1. Introduction

Starfish, a voracious generalist predator, feeds on commercial shellfish (e.g., oysters, cockles, scallops, other clams) [1] and live corals [2], and are extremely reproductive. Thus, their proliferation can be a significant threat to aquaculture and marine ecosystems. So far, Indonesia, Japan, Korea, Australia and other coastal countries have experienced different levels of starfish outbreaks [3,4,5,6]. The potential impact of starfish on native assemblages and commercial species has been a major concern for natural resource managers [1]. Unfortunately, measures such as manual removal, chemical injection of starfish, fencing next to corals, or cutting the bodies of starfish into smaller pieces are not effective in controlling starfish populations [7].

China is rich in starfish resources, with about 50–60 species distributed in Shandong, Zhejiang, Fujian, Guangdong and Nansha Islands and other coasts [8]. *Asterias amurensis* is an extremely common starfish along the Yellow Sea coast of China, which belongs to the marine invertebrates Echinodermata phylum [9]. From 2020–2022, the outbreak of multiple *A. amurensis* floods had a huge impact on the local shellfish farming industry. The mechanism of *A. amurensis* outbreaks is very complex and elusive. Till now, there is no proper solution for its flooding. As an alternative of *A. amurensis* population control, an integrated research for utilizing *A. amurensis* should be conducted. Existing researches show that Starfish contain a variety of active substances such as saponins, polysaccharides, peptides, amino acids, collagen and alkaloids [10], which have potential medicinal value.

Collagen account for approximately 30% of the total protein content of the body, To date, 28 types of collagen have been identified and described [11]. Terrestrial animals (e.g., cattle, sheep, pigs, etc.) serve as the main traditional source of collagen. At present, considering the economic benefits, collagen for industrial purpose is mainly extracted from bovine and porcine origins [12]. However, compared to terrestrial sources, marine sources of collagen have the following advantages: 1. are not subject to religious ethical restrictions, 2. have a lower risk of transmitting diseases [13,14,15], and 3. can be obtained from marine animal waste, which is cost effective and environmentally friendly. Therefore, collagen of marine origin have received increasing attention [16].

Collagen were isolated from the body wall of Tokyo *A. amurensis* as early as 1973 [17]. Since then, *A. amurensis* collagen (AAC) has been studied by an increasing number of scholars. Ka-jeong extracted type I collagen from *A. amurensis* in the coast of Yeosu, Jeonnam, Korea and the collagen did not exhibit any biocompatibility problems with human dermal cells [18]. Studies have also demonstrated that AAC-related incubation enzymes can be used in the cosmetic industry [19]. Some progress has been made in the study of AAC, However, only a few studies have been undertaken on the molecular characterization of collagen derived from the AAC. The objective of this research is to investigate the preparation, biochemical characteristics of collagen from *A. amurensis* from Qingdao, China, and its potential use with BMSCs.

## 2. Results

### 2.1. Protein Pattern of Collagen

**Figure 1:**
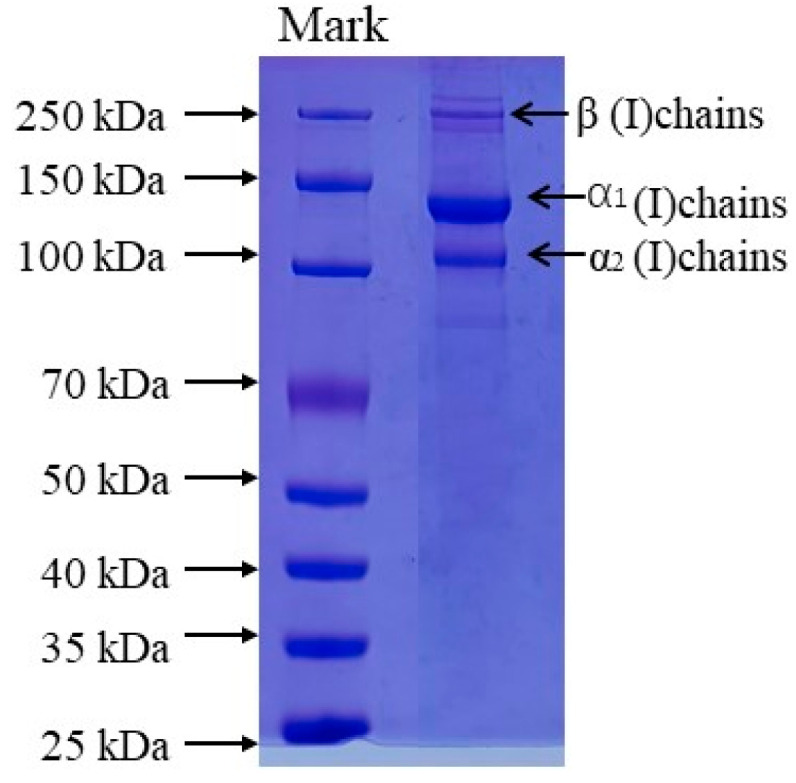
SDS-PAGE patterns of collagen from the skin of starfish. Lane 1: protein markers; lane 2: collagen from starfish.

The protein patterns of AAC were analyzed by SDS-PAGE as shown in Figure 1, The protein pattern of AAC is similar to that of type I collagen, with α_1_, α_2_ and β bands, the molecular weights of α1, α2 and β are about 135, 120 and 250 kDa.

### 2.2. Amino Acid Composition of AAC

The total amino acid residues present in collagen were determined to understand the pattern of amino acid composition in AAC. As shown in Table 1, Starfish collagen contain 6 essential amino acids (valine, isoleucine, leucine, phenylalanine, threonine, lysine), which content of total amino was 120.53 residues/1000 residues. Glycine was the major amino acid (297.43 residues/1000 residues) followed by hydroxyproline (99.80 residues/1000 residues) and alanine (98.81 residues/1000 residues).

### 2.3. Characteristic Absorption of AAC

#### 2.3.1. UV Spectral Properties

**Figure 2:**
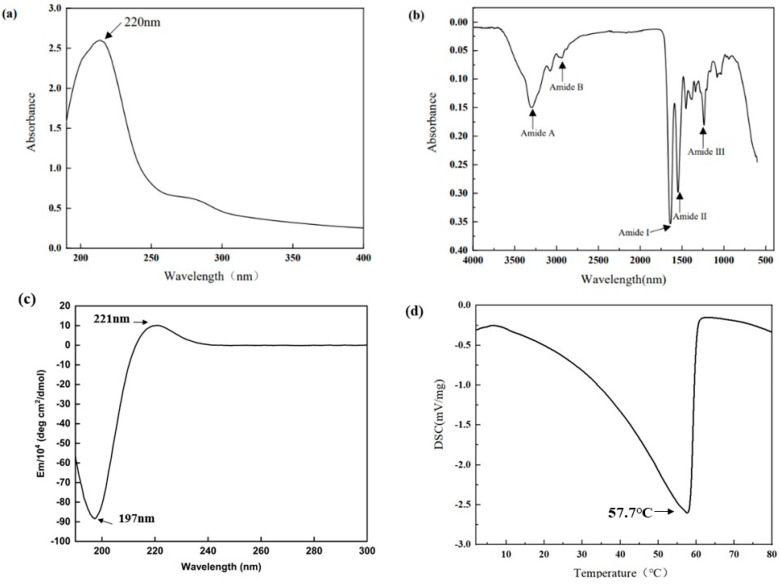
(**a**) FTIR spectrum; (**b**) UV spectrum; (**c**) CD Spectral; (**d**) DSC.

The UV–visible spectrum of collagen sample is depicted in Figure 2a, which shows a highest peak at 220 nm. A small absorption peak was also observed at 280 nm.

#### 2.3.2. FTIR Spectral Properties

The positions of the major peaks in the FTIR spectrum of AAC are shown in Figure 2b. The infrared spectrogram of collagen consist of amides A and B with amides I, II and III. The N–H stretching vibration of the amide A band ranges from 3400–3450 cm^−1^, and this peak correlates with the stretching vibration of N-H in collagen and the hydrogen bonds formed by hydroxyl groups. The amide B band has a stretching vibration range of about 2900 cm^−1^ for C–H. The amide A band of AAC was located at 3310 cm^−1^ and The amide B band was located at 2932 cm^−1^. The C=O stretching vibration range of amide I band and peptide chain is 1600 cm^−1^–1670 cm^−1^, and the C–N stretching vibration and N–H bending vibration range of amide II band is 1500 cm^−1^–1600 cm^−1^. The C–N stretching and N–H bending vibrations of the amide III band ranged from 1200 cm^−1^ to 1360 cm^−1^. The amide I, II and III bands of AAC are located at 1647 cm^−1^, 1543 cm^−1^ and 1233 cm^−1^.

#### 2.3.3. Circular Dichroism (CD) Spectral Properties

CD spectra can reflect the stereoscopic structural information of collagen. The CD spectrum of AAC in the far UV region (190–300 nm) is shown in Figure 2c, with positive absorption peaks at 221 nm and negative absorption peaks at 197 nm.

### 2.4. Thermal Stability

As shown in Figure 2d, there is a thermal peak in AAC. This peak indicates the melting temperature of AAC, which reflects the thermal stability of collagen. The melting temperature of AAC is 57.7 °C.

### 2.5. Microstructural Analysis

**Figure 3:**
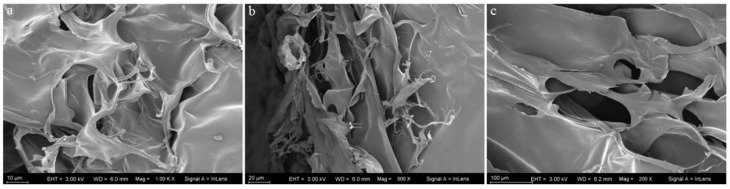
Scanning electron microscopic structural image with different magnifications: (**a**) (10 µm), (**b**) (20 µm), (**c**) (100 µm).

As shown in Figure 3, The microstructural features of collagen are determined by using SEM at different magnifications (100, 20 and 10 µm). It was seen that the collagen had a loose, fibrous, and porous structure.

### 2.6. AAC Promotes BMSCs Proliferation

**Figure 4:**
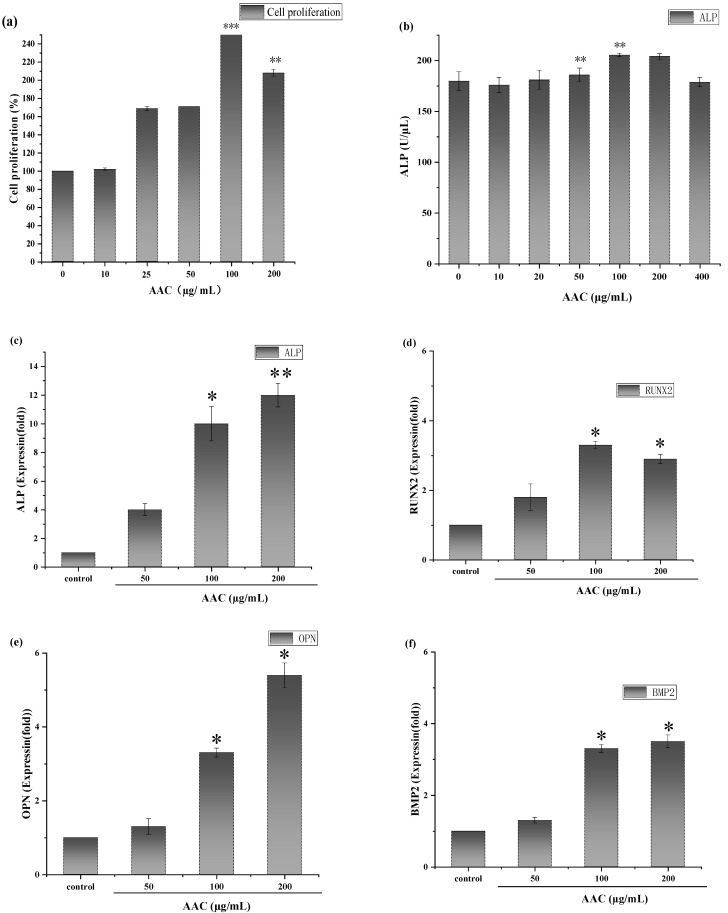
(**a**) Effect of different concentrations of collagen on the proliferative capacity of BMSCs; (**b**) Effect of different concentrations of collagen on ALP activity; Effect of different concentrations of collagen on the relative expression of ALP gene (**c**), RUNX-2 gene (**d**), OPN gene (**e**) and BMP2 gene (**f**). * *p* < 0.05; ** *p* < 0.01; *** *p* < 0.001.

As shown in Figure 4a, consider the cellular value-added rate of the control group as 100%, AAC had a proliferative effect on BMSCs after 48 h in the concentration range of 10–100 µg/mL and the maximum value-added rate (257%) was observed at the concentration of 100 µg/mL (*p* < 0.001). At a concentration of 200 µg/mL, the cell proliferation rate showed a decreasing trend.

### 2.7. AAC Enhances ALP Activity

After 7 days of continuous incubation, ALP activity was measured according to the instructions of ALP kit. As shown in Figure 4b, 50 and 100 µg/mL collagen significantly increased ALP activity in BMSCs compared to the control group (*p* < 0.01).

### 2.8. AAC Promotes Calcified Nodules in BMSCs

**Figure 5:**
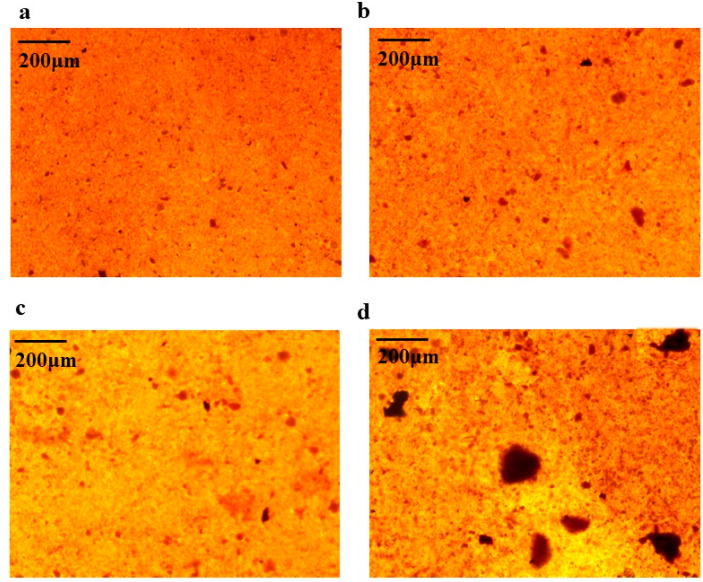
Effect of AAC on the generation of calcified nodules in BMSCs cells. (**a**) Induction for 7 days with induction solution; (**b**) Induction for 7 days with induction solution and collagen; (**c**) Induction for 16 days with induction solution; (**d**) Induction for 16 days with induction solution and collagen.

Alizarin red staining was performed on BMSCs cells on day 7 and day 16 of osteogenesis induction, respectively. Alizarin red forms a deep red complex by chelating calcium ions in mineralized nodules of osteoblasts. Based on the results of 2.7, 100 µg/mL of AAC was chosen for the experiment. As shown in Figure 5, the number and area of calcified nodules in BMSCs cells were significantly increased at days 7 and 16 of induction compared to the control group (*p* < 0.05, *p* < 0.01).

### 2.9. AAC Upregulates mRNA Expression of Osteogenic Genes

To further evaluate the role of AAC in osteogenic differentiation, the effect of collagen on osteogenic differentiation of BMSCs cells was assessed by detecting the relative expression of the genes ALP, Runt-related transcription factor 2 (RUNX2), Osteopontin (OPN) and Bone morphogenetic protein-2 (BMP2).

The results are shown in Figure 4. As shown Figure 4c–f, AAC significantly upregulated the expression of all four genes in the concentration of 100–200 ug/mL, which gave a clue that AAC promoted osteogenic differentiation of BMSCs cells related to these signal pathways. The bioactive mechanism need further elucidation.

## 3. Discussion

### 3.1. SDS-PAGE

The AAC was shown to be compatible with type I collagen and of high purity from SDS-PAGE protein profiles, which also resembled the peptide pattern of collagen in the body wall of crown-of-thorns starfish (*Acanthaster planci*) [20] and starfish (*Asterias rubens*) [21]. 

### 3.2. Analysis of Amino Acid Composition

The molecular structure of collagen is mainly closely related to the changes of the polypeptide chain in the secondary structure. The pyrrole ring in proline can prevent the changes in the secondary structure of the peptide chain, thus enhancing the stability between collagen molecules. The Pro content in AAC is higher than that of common starfish (*Asterias rubens*) [22]. The glycine content is higher than that in Starfish (*Acanthaster planci*) [20]. Hydroxyproline has been found to be a signature amino acid for collagen [23]. Higher levels of proline (86.22 residues/1000 residues) and hydroxyproline (99.80 residues/1000 residues) help stabilize junctions of the collagen structure by forming hydrogen bonds, contributes to the structural stability of the collagen triple helix [24]. Based on the above results, the amino acid composition of starfish collagen is consistent with typical collagen characteristics. The higher the content of Pro, the higher the stability of the helix and the more stable the structure of collagen.

### 3.3. Characteristic Absorption

The UV absorption peak of collagen is generally in the range of 220–250 nm, which may be related to the groups C=O, –COOH, CONH_2_ in the collagen polypeptide chain [16]. The weak absorption at 280 nm in the UV spectrum of AAC may be due to the presence of tyrosine [25].

Based on the results of FTIR, The amide A band of starfish collagen was located at 3310 cm^−1^, which means that the NH groups of the collagen sample is combined with hydrogen bonding. This helps to stabilize the triple helical structure of collagen [20]. This result was similar to the FTIR spectrum of collagen from starfish (*Acanthaster planci*) [20] and blacktip reef shark [25]. The complete triple helix structure of AAC was also confirmed by CD spectral. Also, the triple helix structure of collagen determines its thermal stability [26].

### 3.4. Osteogenic Activity Analysis

The release of ALP from BMSCs occurs at an early stage of osteoblast differentiation, and the secretion of ALP promotes the binding of collagen to the extracellular matrix to form calcified nodules [27]. In the late stage of osteogenic differentiation of BMSCs, the cells release calcium ions through matrix vesicles, which are precipitated on collagen fibrils by ALP, completing the process of matrix mineralization. The mineralized nodules are a sign of mature osteoblast differentiation [28]. ALP is a typical protein product of osteoblast phenotype and osteoblast differentiation. High expression of ALP is a specific marker of osteogenic differentiation and plays a key role in bone development. RUNX2 is an important regulator of osteoblast differentiation and bone formation, which can regulate the expression of osteoblast-specific extracellular mechanism protein genes and osteogenic cycle, thus participating in the process of osteogenic differentiation. OPN is mainly expressed in the early stages of bone formation, it is secreted by cells and stored in the bone matrix, binding itself to the bone matrix, which leads to the maturation of the mineralized bone matrix, and also regulates the formation of transosseous apatite, participates in the deposition of bone salts and promotes mineralized tissue remodeling. BMP2 has the ability to induce undifferentiated mesenchymal stem cells to chondrogenic and osteoblastic cells for directed and promote osteoblast differentiation and maturation [29]. The experimental results demonstrated that AAC could promote osteogenic differentiation of BMSCs.

Bone health plays a vital role in the overall well-being of our life. Reduced bone mineral density and reduced fracture healing ability are associated with reduced osteoblast differentiation and proliferative capacity [30]. Plus, naturally sourced collagen is safer and has fewer side effects. AAC maybe have a potential application in dietary supplements and functional foods to maintain bone health.

## 4. Materials and Methods

### 4.1. Reagents

Sodium hydroxide (NaOH), acetic acid, sodium chloride (NaCl), hydrochloric acid (HCl), ethanol, and potassium bromide (KBr) were purchased from Sinopharm Chemical Reagent (Shanghai, China). Pepsin from porcine stomach mucosa (1:3000 U) was purchased from Beijing Solarbio AACience. Trypsin (1:250), dithiothreitol (DTT), and Coomassie brilliant blue R-250 were purchased from Sigma-Aldrich Corporation (USA). Dual Color protein standard marker with MW of 37–250 kDa (Catalog No. 1610374), 4 × Laemmli Sample Buffer (Catalog No. 1610747), 10 × Tris/Glycine/SDS (Catalog No. 1610732) were purchased from Bio-Rad Laboratories Inc. (Hercules, CA, USA). All reagents were used at an analytical grade unless otherwise specified.

### 4.2. Raw Materials and Pre-Treatment

*Asterias amurensis* were collected in Jiaozhou Bay, Qingdao, China, in 2022 and frozen at −20 °C. After thawing, the starfish guts were removed and the skin was washed with deionized water. They were cut into small pieces (0.5 × 0.5 cm^2^) and stored at −20 °C.

### 4.3. Extraction of AAC

**Figure 6:**
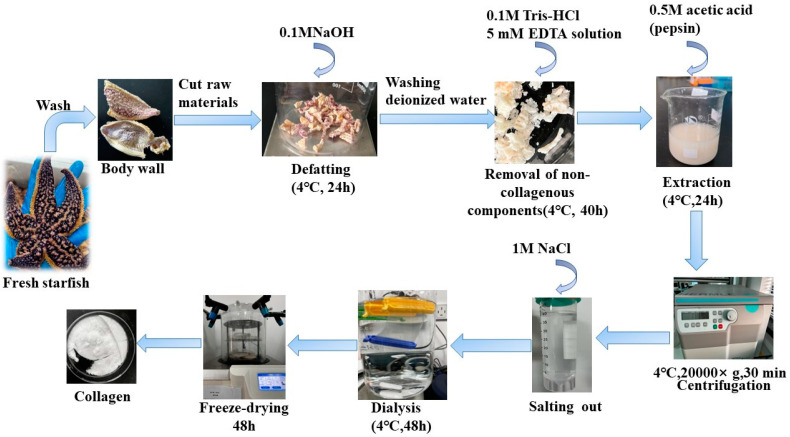
Schematic representation of steps involved in collagen extraction.

Pre-treated Starfish were mixed with 0.1 M NaOH and stirred for 24 h. The alkaline solution was replaced every 8 h to remove fat and pigments. After degreasing, the Starfish were washed to neutral with deionized water, and 0.1M Tris-HCl and 5 mM EDTA solution were added and stirred for 40 h, the solution was replaced every 8 h to remove non-collagenous components such as calcium and heteropolysaccharides. After degreasing and decalcifying, pepsin (0.5% of the starfish) was added and acetic acid solution at a concentration of 0.5 M was added at a 1:10 (*w/v*) ratio of material to liquid, which was stirred for 24 h. Collagen were precipitated with 1 M NaCl, The mixture was then centrifuged at 20,000× *g* for 30 min at 4 °C using a Himac CR 21G High-speed floor centrifuge (Hitachi, Tokyo, Japan). The salting-out precipitates were redissolved in 10 volumes of 0.5 M acetic acid and dialyzed using dialysis membranes (MWCO: 10 kDa) against distilled water until a neutral pH was obtained. Finally, The dialyzed sample was lyophilized by using a freeze-dryer (Labconco Freezone 2.5 L, Kansas City, MO, USA), The collagen extraction yield obtained by this extraction method was 6.82%. A detailed flow chart of the AAC preparation procedure is shown in Figure 6.

### 4.4. Amino Acid Composition

The amino acid content of collagen samples was analyzed by using an amino acid analyzer (Hitachi LA-8080, Tokyo, Japan) [31]. The freeze-dried collagen was completely hydrolyzed in 6 M HCl at 110 ℃ for 24 h. The excess amount of solvent was evaporated under the vacuum incubator. Then, the dried sample was dissolved in distilled water and the drying process was repeated three to four times. In the end, the dried sample was dissolved with a minimum amount of sodium citrate buffer solution (pH 2.2) and filtered through a 0.45 nm hydrophilic membrane. The amino acid analyzer was calibrated with a standard reagent, and a positive control of all amino acids was run as a reference before analyzing the test sample. The retention time of each amino acid peak was equalized with the respective positive control amino acid peak and the content of amino acid was expressed as the number of residues/1000 residues.

### 4.5. Sodium Dodecyl Sulfonate-Polyacrylamide Gel Electrophoresis (SDS-PAGE)

According to Laemmli’s method [32], the molecular pattern of purified collagen was determined by using SDS-PAGE. The collagen samples were mixed with (3:1) 4 × Sample Buffer (SDS, Tris-HCl, bromophenol blue, glycerol, and DTT) and oscillated slightly with a scroll oscillator. The samples were boiled for 5 min and the boiled mixture was briefly centrifuged at 5000 r/min. The test samples and standard protein standard marker (MW ranging from 37 kDa to 250 kDa) were loaded onto 4.5% stacking polyacrylamide gel with 7.5% separating gel. The electrophoresis unit was set at a constant voltage of 200 V in order to efficiently separate the collagen samples using a mini-PROTEAN Tetra Cell. After the electrophoresis, the gel was stained with 0.25% Coomassie brilliant blue R-250 solution for 30 min and discolored with the mixture of 20% (*v/v*) ethanol and 10% (*v/v*) acetic acid twice, each for 1 h until clear protein bands were observed. The protein bands were then captured using the gel documentation system.

### 4.6. Ultraviolet (UV) Scanning Analysis

The collagen were dissolved in a 0.5 M acetic acid solution, and the concentration was adjusted to 1 mg/mL. The sample was then scanned using UV spectrophotometer (MAPADA UV-3000PC, Shanghai, China). The scanning range was from 200 to 400 nm and the scanning rate was 1 nm/s. The baseline was calibrated with a solution of 0.5 M acetic acid.

### 4.7. Fourier-Transform Infrared (FTIR) Spectroscopy

The collagen was mixed with the same amount of KBr and grinded into fine powder. The sample was pressed and observed using FTIR spectroscopy (PerkinElmer, Waltham, MA, USA). The scanning wavelength ranges were from 500 cm^−1^ to 4000 cm^−1^, and the signals were collected in 64 scans at a resolution of 4 cm^−1^.

### 4.8. CD Spectral

An appropriate amount of lyophilized AAC was weighed and dissolved in 0.5 M acetic acid solution to make the final concentration of AAC solution 0.2 mg/mL, which was injected into a 1 mm quartz cell, and the sample was scanned at 190–300 nm using circular dichroism chromatograph (JASCO J-1500, Tokyo, Japan) with a scan rate of 100 nm/min and a width of 1 nm, and acetic acid was used as a blank control, and the scan was repeated five times to obtain the average value.

### 4.9. Scanning Electron Microscopy (SEM)

The collagen structure was observed by SEM (Hitachi SU5000, Tokyo, Japan). Freeze-dried collagen was cut into suitable pieces and fixed on the scaffold of the microscope. The microstructure of the sample was observed after vacuum ion spraying.

### 4.10. Differential scanning Calorimetry (DSC)

Thermal stability analysis of samples by differential scanning calorimetry (TAQ 2000, Hercules, CA, USA). The collagen was dried under vacuum before characterization. The experiments were carried out under N_2_-filled conditions with a nitrogen flow rate of 25 mL/min and a temperature increase rate of 2 °C/min. The temperature was measured in the range of 2 ℃ to 80 ℃.

### 4.11. Cell Culture

BMSCs were obtained from the scienCell, Beijing, China; cultivated in MEM medium containing l-glutamine and sodium bicarbonate and supplemented with 10% FBS and 1% penicillin/streptomycin, respectively. The cells were grown in 90 mm petrie dish in a regulated CO_2_ incubator (HMUSSE, SQ-80N, Shanghai, China) at 37 ℃. Cells were confluent to 80–90%, washed several times with PBS (pH 7.4), digested with 0.25% trypsin and used for counting, plate spreading.

### 4.12. Proliferation Assay

The BMSCs in logarithmic growth phase were inoculated in 96-well plates at 1 × 10^4^/well, and after cell walling, 100 µL of collagen solution (10, 25, 50, 100, 200 µg/mL) was added, and the control group was incubated for 48 h with drug-free medium [33]. The cells were incubated for 48 h with 10 µL CCK-8 and incubated for 2 h. The absorbance values at 450 nm were measured by Enzyme Markers (HBS-1096A, Nanjing, China).

### 4.13. Alkaline Phosphatase (ALP) Activity

The BMSCs were cultured for one day and then inoculated at the cells were inoculated in 12-well plates at a density of 1 × 10^4^/well. When the cells were close to fusion, the osteogenic induction medium (containing 1 × 10^−8^ mol/L dexamethasone, 10 mmol/L sodium β-glycerophosphate, 50 mg/L ascorbic acid) was replaced with 1 mL of collagen solution (10, 25, 50, 100, 200 µg/mL), and the control medium without drugs was added, and the induction medium was changed every 3 days. After 7 days of continuous incubation, ALP activity was measured according to the instructions of ALP kit (TIANGEN, Beijing, China).

### 4.14. Calcified Cell Nodules

The BMSCs were cultured for one day and then inoculated at the cells were inoculated in 12-well plates at a density of 1 × 10^4^/well, and the cells were grown close to fusion. When the cells were close to fusion, the osteogenic induction medium (containing 1 × 10^−8^ mol/L dexamethasone, 10 mmol/L sodium β-glycerophosphate, 50 mg/L ascorbic acid) was replaced with 1 mL of collagen solution (10, 50, 100 µg/mL), and the control medium without drugs was added, and the induction medium was changed every 3 days and incubated for 17 days, observe the alizarin red staining by the light microscope (XSP-H1600, Shenzhen, China).

### 4.15. RT-PCR Detection of Genes Related to Osteogenic Differentiation in Mice

Briefly, total RNA was extracted from cells using the Total RNA Extraction Kit (TIANGEN, Beijing, China). After purity testing, total RNA was synthesized into cDNA using the FastKing cDNA First Strand Synthesis Kit (TIANGEN, Beijing, China). Quantitative real-time PCR was performed using the Talent Fluorescence Quantification Kit (SYBR Green) (TIANGEN, Beijing, China) and the 7500 Fast Real-Time PCR System (Applied Biosystems, CA, USA). The program was set to run for one cycle at 95 °C for 5 min, 40 cycles at 95 °C for 10 s and at 60 °C for 30 s. Calculate the relative expression of relevant genes. The primer sequences used are shown in the Table 2.

### 4.16. Statistical Analysis

SPSS 19.0 software was used for statistical and analysis, and the data of each group was the data of each group were expressed by x ± s. One-way ANOVA was used for comparison between groups, and a Tukey’s test was used for post hoc multiple comparisons. A value of *p* < 0.05 was considered statistically significant. * *p* < 0.05; ** *p* < 0.01; *** *p* < 0.001.

## 5. Conclusions

Collagen, as an essential organic substance in the body, plays a vital role in several life activities. Bone tissue is composed of 1/3 organic and 2/3 inorganic substances, with type I collagen accounting for about 80–90% of the organic component. It is very important for maintaining the integrity of bone structure and bone biology [34]. Osteoporosis is a systemic metabolic bone disease characterized by low bone mass, microarchitectural changes, and increased bone fragility. Changes in collagen structure and quantity are closely related to the onset, progression and severity of osteoporosis [35]. It has been shown that collagen slows the process of osteoporosis by regulating the balance between bone formation and bone resorption [35].

In this study, type I collagen was firstly extracted from Qingdao starfish (*Asterias amurensis*), which had a stable triple helix structure. Additionally, its osteogenic differentiation ability on BMSCs was explored. In vitro experiments demonstrated that AAC promotes the proliferation of BMSCs and promotes osteogenic differentiation of cells by increasing the activity of ALP, enhancing cell mineralization and upregulating the expression of osteogenic factor mRNA. The results of these studies can provide useful strategic references for the development of functional foods with collagen.

## Figures and Tables

**Table 1:** Amino acid composition of AAC (residues/1000 residues).

Amino Acid	Starfish Collagen
Aspartic acid (Asp)	82.51 ± 0.92
Threonine (Thr)	32.36 ± 0.37
Serine (Ser)	68.66 ± 1.51
Glutamic acid (Glu)	89.42 ± 0.25
Glycine (Gly)	297.43 ± 0.58
Alanine (Ala)	98.81 ± 3.59
Valine (Val)	21.98 ± 0.37
Isoleucine (Ile)	15.06 ± 0.38
Leucine (Leu)	16.30 ± 0.38
Tyrosine (Tyr)	8.40 ± 0.38
Phenylalanine (Phe)	8.89 ± 0.38
Lysine (Lys)	25.94 ± 0.21
Histidine (His)	2.22 ± 0.2
Arginine (Arg)	45.95 ± 0.42
Proline (Pro)	86.22 ± 0.25
Hydroxyproline (Hyp)	99.80 ± 0,37
Cysteine (Cys)	0
Total	1000

Data are expressed as mean ± standard deviation, *n* = 3.

**Table 2:** Primers used for RT-PCR analysis.

Gene	PCR Primer	
	F	R
GAPDH	5′-AATCCCATCACCATCTTCC-3′	5′-GCAGAGATGATGACCCTTT-3′
ALP	CACGGCGTCCATGAGCAGAAC	CAGGCACAGTGGTCAAGGTTGG
RUNX-2	CGGCAAGATGAGCGACGTGAG	GCTGTTGTTGCTGCTGCTGTTG
OPN	ATGGACGACGATGATGACGATGATG	CTTGTGTACTAGCAGTGACGGTCTC
BMP2	AAGCGTCAAGCCAAACACAAACAG	GAGGTGCCACGATCCAGTCATTC

## Data Availability

The data presented in this study are available on request from the corresponding author.

## References

[1] Jeff Ross D., Johnson C.R., Hewitt C.L. (2003). Variability in the impact of an introduced predator (*Asterias amurensis*: Asteroidea) on soft-sediment assemblages. J. Exp. Mar. Biol. Ecol..

[2] Brodie J., Fabricius K., De’ath G., Okaji K. (2005). Are increased nutrient inputs responsible for more outbreaks of crown-of-thorns starfish? An appraisal of the evidence. Mar. Pollut. Bull..

[3] Reichelt R.E., Bradbury R.H., Moran P.J. (1990). The crown-of-thorns starfish, *Acanthaster planci*, on the great barrier reef. Math. Comput. Model..

[4] Ross D.J., Johnson C.R., Hewitt C.L.J.B.I. (2003). Assessing the ecological impacts of an introduced seastar: The importance of multiple methods. Biol. Invasions.

[5] Hatanaka M., Kosaka M. (1959). Biological studies on the population of the starfish, *Asterias amurencis*, in Sendai Bay. Tohoku J. Agr. Res..

[6] Nojima S., Soliman F.E., Kondo Y., Kuwano Y., Nasu K., Kitajima C. (1986). Some notes of the outbreak of the sea star *Asterias amurensis* versiclor Sladen, in the Ariake Sea, western Kyshu. Publ. Amakusa Mar. Biol. Lab. Kyushu Univ..

[7] Fraser N., Crawford B.R., Kusen J., Indonesia P.P.C. (2000). Best Practices Guide for Crown-of-Thorns Clean-Ups.

[8] Mah C.L., Blake D.B. (2012). Global diversity and phylogeny of the Asteroidea (Echinodermata). PLoS ONE.

[9] Grannum R., Murfet N., Ritz D., Turner E. (1996). The Distribution and Impact of the Exotic Seastar, Asterias amurensis (Lutken) in Tasmania.

[10] Malyarenko T.V., Kicha A.A., Ivanchina N.V., Kalinovsky A.I., Popov R.S., Vishchuk O.S., Stonik V.A. (2014). Asterosaponins from the Far Eastern starfish *Leptasterias ochotensis* and their anticancer activity. Steroids.

[11] Salvatore L., Gallo N., Natali M.L., Campa L., Lunetti P., Madaghiele M., Blasi F.S., Corallo A., Capobianco L., Sannino A. (2020). Marine collagen and its derivatives: Versatile and sustainable bio-resources for healthcare. Mater. Sci. Eng. C.

[12] Jongjareonrak A., Benjakul S., Visessanguan W., Nagai T., Tanaka M. (2005). Isolation and characterisation of acid and pepsin-solubilised collagens from the skin of Brownstripe red snapper (*Lutjanus vitta*). Food Chem..

[13] Kozlowska J., Sionkowska A., Skopinska-Wisniewska J., Piechowicz K. (2015). Northern pike (*Esox lucius*) collagen: Extraction, characterization and potential application. Int. J. Biol. Macromol..

[14] Ahmed M., Verma A.K., Patel R. (2020). Collagen extraction and recent biological activities of collagen peptides derived from sea-food waste: A review. Sustain. Chem. Pharm..

[15] Hou Y., Shavandi A., Carne A., Bekhit A.A., Ng T.B., Cheung R.C.F., Bekhit A.E.-d.A. (2016). Marine shells: Potential opportunities for extraction of functional and health-promoting materials. Crit. Rev. Environ. Sci. Technol..

[16] Veeruraj A., Arumugam M., Balasubramanian T. (2013). Isolation and characterization of thermostable collagen from the marine eel-fish (*Evenchelys macrura*). Process Biochem..

[17] Matsumura T. (1973). Shape, size and amino acid composition of collagen fibril of the starfish *Asterias amurensis*. Comp. Biochem. Physiol. Part B Comp. Biochem..

[18] Lee K.-j., Park H.Y., Kim Y.K., Park J.I., Yoon H.D. (2009). Biochemical Characterization of Collagen from the Starfish *Asterias amurensis*. J. Korean Soc. Appl. Biol. Chem..

[19] Li Z.J., Kim S.M. (2014). Structural Identification and Proteolytic Effects of the Hatching Enzyme from Starfish *Asterias amurensis*. Protein Pept. Lett..

[20] Tan C., Karim A., Latiff A., Gan C., Ghazali F. (2013). Extraction and characterization of pepsin-solubilized collagen from the body wall of crown-of-thorns Starfish (*Acanthaster planci*). Int. Food Res. J..

[21] Kumar Vate N., Pawel Strachowski P., Undeland I., Abdollahi M. (2023). Structural and functional properties of collagen isolated from lumpfish and starfish using isoelectric precipitation vs salting out. Food Chem. X.

[22] Vate N.K., Undeland I., Abdollahi M. (2022). Resource efficient collagen extraction from common starfish with the aid of high shear mechanical homogenization and ultrasound. Food Chem..

[23] Stoilov I., Starcher B.C., Mecham R.P., Broekelmann T.J. (2018). Measurement of elastin, collagen, and total protein levels in tissues. Methods in Cell Biology.

[24] Kaewdang O., Benjakul S., Kaewmanee T., Kishimura H. (2014). Characteristics of collagens from the swim bladders of yellowfin tuna (*Thunnus albacares*). Food Chem..

[25] Ge B., Hou C., Bao B., Pan Z., de Val J.E.M.S., Elango J., Wu W. (2022). Comparison of Physicochemical and Structural Properties of Acid-Soluble and Pepsin-Soluble Collagens from Blacktip Reef Shark Skin. Mar. Drugs.

[26] Song X., Si L., Sun X., Zhu X., Li Z., Li Y., Wang Y., Hou H. (2022). Rheological properties, thermal stability and conformational changes of collagen from sea cucumber (*Apostichopus japonicas*). Food Chem..

[27] Xie Y.-F., Shi W.-G., Zhou J., Gao Y.-H., Li S.-F., Fang Q.-Q., Wang M.-G., Ma H.-P., Wang J.-F., Xian C.J. (2016). Pulsed electromagnetic fields stimulate osteogenic differentiation and maturation of osteoblasts by upregulating the expression of BMPRII localized at the base of primary cilium. Bone.

[28] Tanabe N., Ito-Kato E., Suzuki N., Nakayama A., Ogiso B., Maeno M., Ito K. (2004). IL-1α affects mineralized nodule formation by rat osteoblasts. Life Sci..

[29] Gao S., Chen B., Zhu Z., Du C., Zou J., Yang Y., Huang W., Liao J. (2023). PI3K-Akt signaling regulates BMP2-induced osteogenic differentiation of mesenchymal stem cells (MSCs): A transcriptomic landscape analysis. Stem Cell Res..

[30] Charoensin S., Pothacharoen P., Wanachewin O., Kongtawelert P., Suttajit M., Pandey K.B., Suttajit M. (2023). Chapter 15—Functional foods in improving bone health during aging. Plant Bioactives as Natural Panacea against Age-Induced Diseases.

[31] Kittiphattanabawon P., Benjakul S., Visessanguan W., Shahidi F. (2010). Isolation and characterization of collagen from the cartilages of brownbanded bamboo shark (*Chiloscyllium punctatum*) and blacktip shark (*Carcharhinus limbatus*). Food Sci. Technol..

[32] Laemmli U.K. (1970). Cleavage of structural proteins during the assembly of the head of bacteriophage T4. Nature.

[33] Luo X., Liu W., Zhao M., Liu T., Xiong F., Lei L., Jia F., Feng F. (2022). A novel Atlantic salmon (*Salmo salar*) bone collagen peptide delays osteoarthritis development by inhibiting cartilage matrix degradation and anti-inflammatory. Food Res. Int..

[34] Mallick M., Are R.P., Babu A.R. (2022). An overview of collagen/bioceramic and synthetic collagen for bone tissue engineering. Materialia.

[35] Licini C., Vitale-Brovarone C., Mattioli-Belmonte M. (2019). Collagen and non-collagenous proteins molecular crosstalk in the pathophysiology of osteoporosis. Cytokine Growth Factor Rev..

